# Enhancement of curcumin antitumor efficacy and further photothermal ablation of tumor growth by single-walled carbon nanotubes delivery system *in vivo*

**DOI:** 10.1080/10717544.2019.1672829

**Published:** 2019-10-03

**Authors:** Haixia Li, Nan Zhang, Yongwei Hao, Yali Wang, Shasha Jia, Hongling Zhang

**Affiliations:** School of Pharmaceutical Sciences, Zhengzhou University, Zhengzhou, PR China

**Keywords:** Curcumin, single-walled carbon nanotubes, nanocarriers, photothermal effect, cancer therapy

## Abstract

Curcumin, a commonly used natural product for antitumor therapy, is unable to achieve full potential due to poor bioavailability. Based on our previous report of a novel delivery system for curcumin using functionalized single-walled carbon nanotubes by phosphatidylcholine and polyvinylpyrrolidone (SWCNT-Cur), we further evaluated SWCNT-Cur’s performance *in vivo* and characteristics *in vitro*. SWCNT-Cur significantly increased the blood concentration of curcumin, up to 18-fold, in mice. And in a murine S180 tumor model, SWCNT-Cur exhibited significantly higher inhibition efficacy on tumor growth and no obvious toxicity in main organs. Moreover, photothermal therapy induced by SWCNT under near-infrared radiation further facilitated SWCNT-Cur to inhibit the tumor growth *in vivo*. In addition, solvent residue is negligible in SWCNT-Cur formulation, and hydrogen bonding was formed between void carriers and curcumin, as demonstrated by GC chromatograph and IR spectra. Furthermore, experiments of confocal microscopy and spectrofluorometer showed that SWCNT-Cur gave a six-fold higher uptake for curcumin compared to native curcumin in human prostate cancer PC-3 cells. In conclusion, curcumin delivery with functionalized SWCNT is a promising strategy to enhance anticancer activity *in vivo* by enhancing cell uptake and blood concentration, changing physicochemical properties of curcumin and combining phototherapeutic with chemotherapeutic effects.

## 1. Introduction

Curcumin (Cur), a hydrophobic polyphenol compound extracted from the rhizome of *Curcuma longa* L., has exhibited extensive anti-tumor potential in a wide range of cancer, such as breast, colon, colorectal, pancreatic, prostate, esophageal, head and neck cancers, endometrial carcinoma, and glioblastoma multiforme (El Khoury et al., 2019; Forouzanfar et al., 2019; Karavasili et al., 2019; Komal et al., 2019; Shahcheraghi et al., 2019; Yeung et al., 2019). Moreover, Cur can inhibit the invasion and metastasis of various cancers (Li & Zhang, 2014; Shafabakhsh et al., 2019; Wang et al., 2019). Furthermore, Cur has demonstrated inhibitory effects on both the expression and function of multidrug resistance proteins (Lin et al., 2018), and has showed synergistic effects with chemotherapy (Lin, et al., 2018; Tan & Norhaizan, 2019). In addition, Cur is cheap and easily available (Willenbacher et al., 2019). Clinically, it is considered extremely safe while administered at doses as high as 8 g/day without dose-limiting toxicities (Kanai et al., 2011). Therefore, the administration of Cur in cancer treatment has been suggested by numerous research groups around the globe (Panda et al., 2017; Tan & Norhaizan, 2019; Vallee et al., 2019; Willenbacher, et al., 2019). However, the poor bioavailability, which makes it not to reach the blood concentration required for anticancer efficacy in patients, remains the major barriers in Cur’s clinical application. Therefore, various attempts, including development of better formulations, become one of the main efforts so as to harness the potential of Cur (Jamwal, 2018; Zheng et al., 2018; Saheb et al., 2019; Wong et al., 2019).

The allotropes of carbon-based nanomaterials (graphene, fullerene, carbon nanotube), have been one of the most promising polymer matrices for their unique physicochemical properties, such as remarkable cell membrane penetrability and extraordinary photothermal effects (Du et al., 2015; Sahne et al., 2019; Shi et al., 2019). Benefits of graphene delivery systems for Cur have been confirmed in cell experiments (Malekmohammadi et al., 2018; Shi, et al., 2019) and in mice bearing subcutaneous 4T1 breast cancer model (Sahne et al., 2019). Similar results *in vitro* have been obtained for single-walled carbon nanotubes (SWCNT). Nanoparticles consisted of Cur and functionalized SWCNT (SWCNT-Cur), firstly prepared by us, induced a significantly higher growth inhibition compared to native Cur and irradiated SWCNT in PC-3 cancer cells, and the inhibitory effect was further significantly enhanced by laser irradiation (Li et al., 2014a). Later, Cur delivery systems based on modified SWCNT by polysaccharides, such as alginate and chitosan, or by different chemical groups, were also reported to display considerable proliferation inhibition efficacy in lung cancer cells or splenic lymphocytes when compared with free Cur (Yuan et al., 2016; Singh et al., 2018). However, *in vitro* activities is not equal to *in vivo* effects, whether SWCNT could increase Cur’s antitumor effects *in vivo* remains unknown.

Herein, *in vivo* evaluation was conducted on SWCNT-Cur for obtaining an exhaustive conclusion about benefits of SWCNT The blood concentration, the antitumor treatment efficacy and the side effect on major organs of SWCNT-Cur were investigated in healthy mice and S180-bearing mice, respectively, and solvent residue, infrared spectrum and cell uptake characteristics of SWCNT-Cur were determined *in vitro.*

## 2. Materials and methods

### 2.1. Materials

SWCNT were purchased from Chengdu Organic Chemicals Co. Ltd. Curcumin was extracted and purified by our laboratory (purity > 98% as determined by HPLC and NMR). Phosphatidylcholine (PC), polyvinylpyrrolidone K30 (PVP K30), and Tween 20 were purchased from Sigma-Aldrich Inc (St Louis, MO).

### 2.2. Cell culture and animals

Human prostate cancer PC-3 cells and S180 mouse ascites tumor cells were purchased from Shanghai Institute of Biochemistry and Cell Biology (Shanghai, China) and Chinese Academy of Sciences Cell Bank, respectively. PC-3 cells were cultured in RPMI-1640 medium, containing 10% of fetal bovine serum, 1 × 10^5^ U·L^−1^ penicillin and 1 × 10^5^ μg · L ^−1^ streptomycin, at 37 °C with 5% CO_2_ in a humidified incubator. Cells at logarithmic growth phase were used.

Healthy male Kunming mice were purchased from Experimental Animal Center of Henan Province. All care and treatment of animals were in accordance with the ‘Guidelines for the Care and Use of Laboratory Animals’ and approved by the Animal Ethics Committee of Zhengzhou University, which follow the guidelines promulgated by the National Act on the treatment of experimental animals (People’s Republic of China).

### 2.3. Preparation of SWCNT-Cur

SWCNT-Cur were prepared as our previously described (Li et al., 2014a). Briefly, pristine SWCNT were treated using sonication and acid reflux to afford short, purified nanotubes and to introduce carboxylic acid groups. Subsequently, Cur was conjugated with SWCNT in methanol containing PVP K30 by ultrasonication. After the mixed solution was dried, the complex was suspended with aqueous solution containing PC and PVP K30 using sonication. After centrifugation, the supernatant solution was collected as SWCNT-Cur.

### 2.4. Solvent residue and Fourier transform infrared (FTIR) spectral analysis

Because methanol was used during SWCNT-Cur preparation, its residual was detected by Agilent 6890 N Gas Chromatography (GC), including a capillary column (30 m × 0.53 mm, 1.0 μm) and flame ionization detector (FID). The carrier gas was nitrogen and the following temperature program was employed: 60 °C (5 min), 60–280 °C at 30 °C/min and 280 °C (5 min). The temperatures of detection and injection port were set at 260 °C and 220 °C, respectively. Split sampling mode was used and the ratio was 100:1. After the sample or methanol was diluted in dimethyl formamide (DMF), 1 mL of the solution was headspace injected to GC.

Further, the FTIR spectra were used to investigate the possible interactions between the Cur and SWCNT carriers. After the samples were mixed and milled with potassium bromide (KBr), they were crushed into the pellets for analysis by FT-IR (Nicolet iS10 spectrometer, Thermo Scientific).

### 2.5. Qualitative and quantitative cellular uptake

After PC-3 cells (1 × 10^5^/well) were seeded in the 6-well plates on cover glasses for 24 h, native Cur and SWCNT-Cur at a final Cur concentration of 40 μM were added, respectively. At designated time points, the cells were rinsed with precooled PBS for 2 times to remove the Cur outside the cells, and were immobilized by acetone, then to image by a laser confocal microscopy.

To further determine the exact amount of Cur delivered into cells by the SWCNT-Cur, PC-3 cells (5 × 10^5^/well) were collected at 4 h after incubation in the presence or absence of 40 μM Cur, rinsed two times with PBS, then sonicated in 3 ml of methanol till Cur is completely extracted. The fluorescent absorption of methanol supernatant containing Cur was determined by a spectrofluorometer (excitation 443 nm/emission 543 nm) after the lysate was centrifuged at 10,000 rpm for 5 min (Kunwar et al., 2007). Next, after deducting fluorescence background, the Cur’s amount was calculated according to the standard curve of Cur: *Y* = 3012.5*x* + 11.914, which were constructed from fluorescence intensity of the Cur versus its concentration.

### 2.6. *In vivo* pharmacokinetics

Mice weighing 20–25 g were randomly divided into two groups with three animals per group. Group 1 was given Cur dissolved in PBS containing 0.1% Tween 20 (Mohanty & Sahoo, 2010); and group 2 was given SWCNT-Cur solution of PBS. Either native Cur or SWCNT-Cur, containing 18.8 mg/kg Cur, was injected *via* lateral tail vein after mouse was fasted for 12 h. Blood samples were collected at designated time points to separate plasma, and the Cur’s concentration was determined by HPLC.

An Agilent 1100 HPLC system (Agilent Technologies, CA) comprising an autosampler, a quaternary solvent delivery system, an online degasser, a column temperature controller and a UV-VIS detector system was used to acquire chromatograms. All analyses were performed using a Waters Atlantis C_18_ Columns (150 mm × 4.6 mm, 3 μm) coupled with C_18_ guard column (10 mm × 4.6 mm). The sample injection volume was 20 μL. The gradient solvent system, which was reported previously by our group (Li et al., 2014b), was employed: 0–15 min, 10–15% methanol, 30–40% tetrahydrofuran, and 45–60% 0.1% phosphoric acid in water. The flow rate was 1.0 ml·min^−1^. The column temperature and detection wavelength were set at 35 °C and 425 nm, respectively.

### 2.7. Animal model and treatment

The S180 tumor models were generated by subcutaneous injection (s.c.) of 2 × 10^7^ S180 cells in 0.2 ml PBS into the right shoulder of male mice. The mice were used for treatment at 10 days after cells inoculation.

All animals were randomized to eight groups with six animals per group. Groups 1, 3, 5, and 7 were control, SWCNT, Cur and SWCNT-Cur, respectively, and the mice received normal saline, void vector, Cur, and SWCNT-Cur, respectively. Groups 2, 4, 6, and 8 were control + laser, SWCNT + laser, Cur + laser, and SWCNT-Cur + laser, respectively, and the mice received 808 nm laser irradiation at 1.4 W/cm^2^ for one minute at the spot of tumor after the same administrations for 5 min as for groups 1, 3, 5, and 7, respectively. All drugs were administered intravenously through lateral tail vein every day, and the injected doses were 18.8 mg/kg of Cur and 6.9 mg/kg of SWCNT. The tumor volumes were measured by a Vernier caliper every day, calculated as the volume = (tumor width)^2^ × (tumor length)/2. And, the tumors were weighed and photographed after the following procedures were performed: euthanizing the mice at designated time point, completely stripping and collecting the tissues of tumor, then sopping up their water with filter paper. The inhibition rate of tumor weight (%) = (1 − average tumor weight of each experimental group/average tumor weight of normal saline group) × 100%

### 2.8. Histological analysis

After seven times of administration, the mice were euthanized, and tissues of heart, liver, spleen, lung, and kidney were collected and soaked in 10% formalin solution. Performing the same process after tumor tissues were weighed and photographed. Then, all tissues were embedded in paraffin, and their slides were stained with hematoxylin–eosin (HE). The morphological changes were evaluated under a microscope.

### 2.9. Statistical analysis

Quantitative data are expressed as mean ± standard deviation (SD). Differences between groups were analyzed using an analysis of variance (ANOVA) followed by Dunnett’s test by SPSS17.0 statistical software, and *p* values of < .05 were considered to be statistically significant.

## 3. Results

### 3.1. Characterization of SWCNT-Cur

#### 3.1.1 Solvent residue

**Figure 1.**
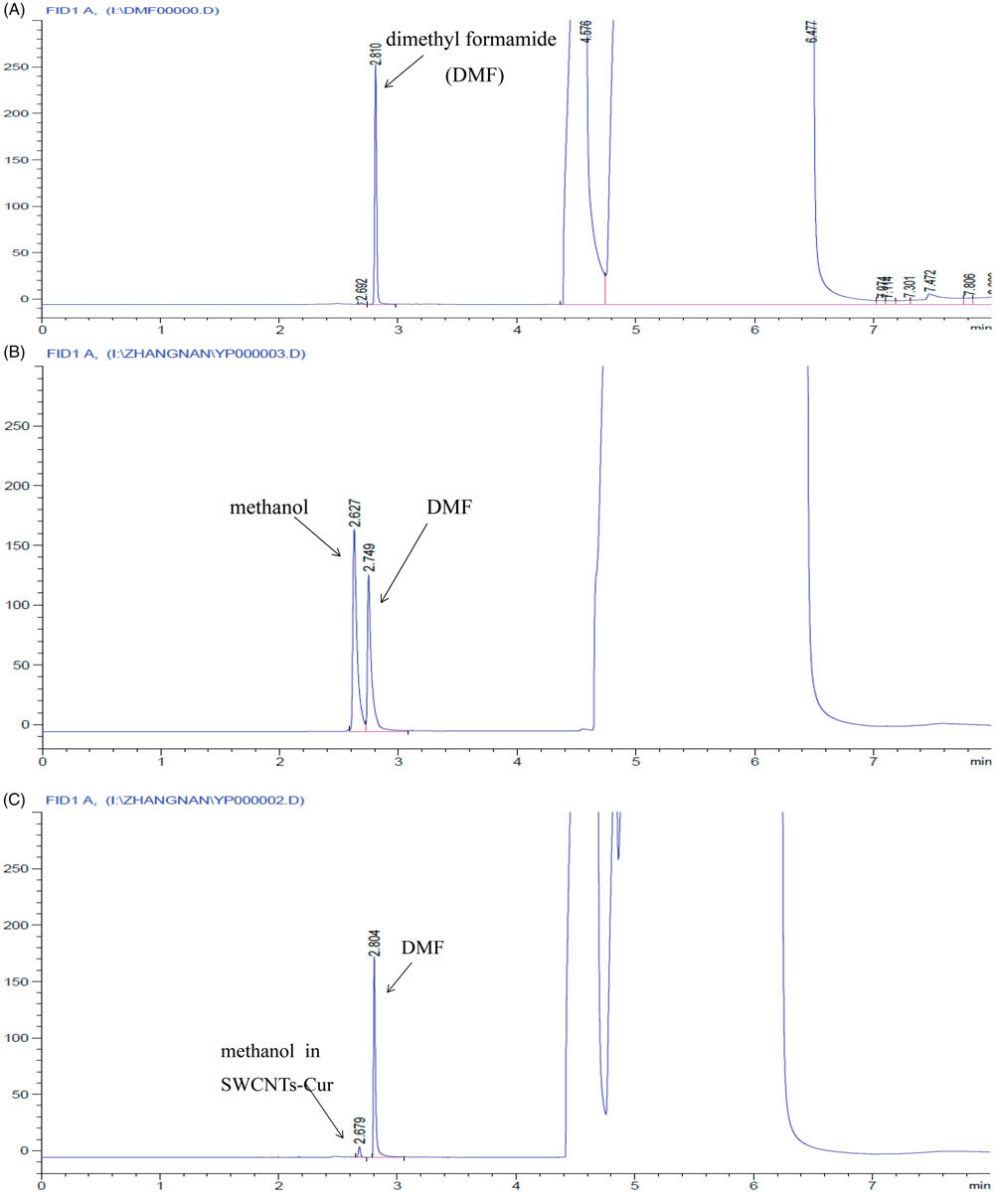
Gas chromatograms of SWCNT-Cur. Dimethyl formamide (DMF) was used to fully dissolve preparation for detecting the residual methanol. (A) DMF, (B) methanol and DMF, (C) SWCNT-Cur and DMF.

The retention time and peak areas in GC between the formulation and the chromatographic grade methanol were compared to identify and quantify methanol. As shown in Figure 1, the peak areas of methanol in formula was far less than the required levels of injectable in the China pharmacopeia 2015 for residual solvent content of methanol (0.3%). Therefore, solvent residue is negligible in SWCNT-Cur formulation.

#### 3.1.2. IR characterization

**Figure 2.**
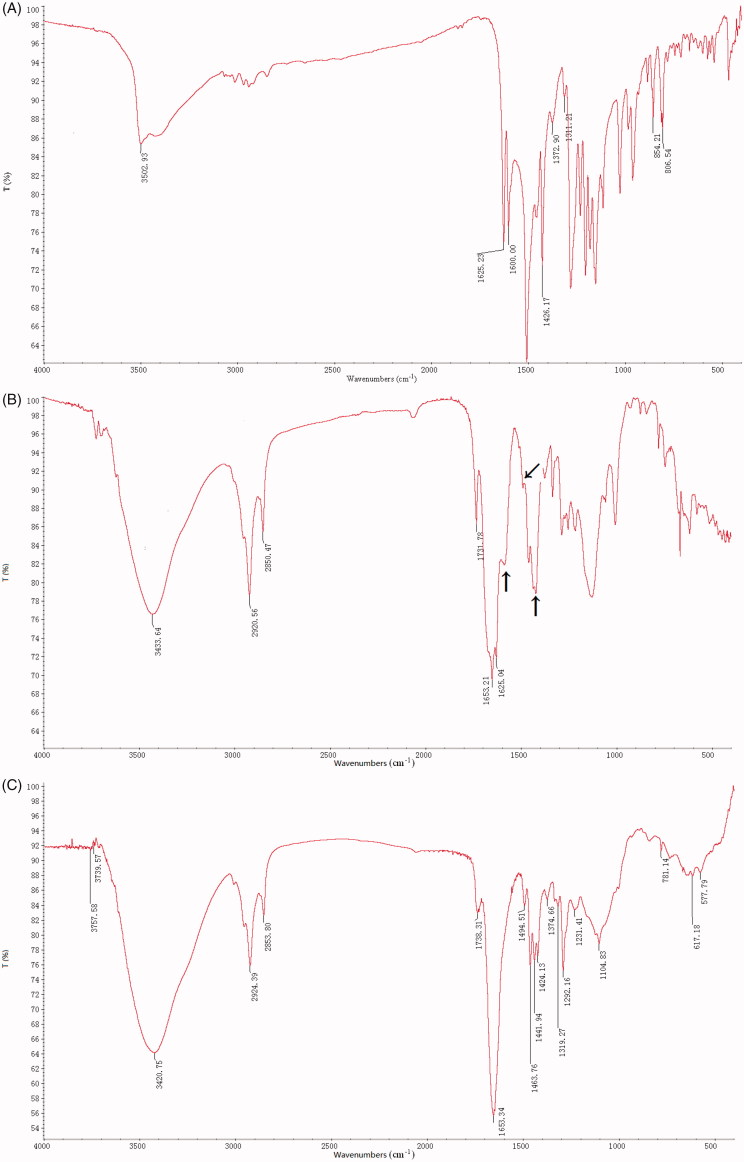
IR spectra of SWCNT-Cur. (A) native curcumin, (B) SWCNT-Cur, (C) void carriers. Arrows indicate the − C = C − conjugated aromatic skeleton vibration of curcumin.

The infrared spectra are shown in Figure 2. A peak at 3502.93 cm^−1^ in native Cur, attributed to − OH stretching vibration, shift to a low frequency of 3433 cm^−1^ in SWCNT-Cur with becoming wider and stronger compared with native Cur, indicating the formation of hydrogen bonds between void carriers and Cur. The strong characteristic peaks at 1600, 1505, and 1426 cm^−1^ (−C = C − conjugated aromatic skeleton vibration), were maintained but became weaker in SWCNT-Cur, suggesting the conjugation strength of − C = C − was reduced due to forming intermolecular hydrogen bonds between Cur and carrier. Besides, a peak at 1625 cm^−1^ in native Cur belongs to the characteristic of stretching vibration of benzene conjugated carbonyl (Ph-C = O). Similar to our results, the maintenances of characteristic peaks of Cur have been described in Cur’s nanoparticulate formulation using glycerol monooleate and pluronic F-127 (Mohanty & Sahoo, 2010).

### 3.2. Cellular uptake study

**Figure 3.**
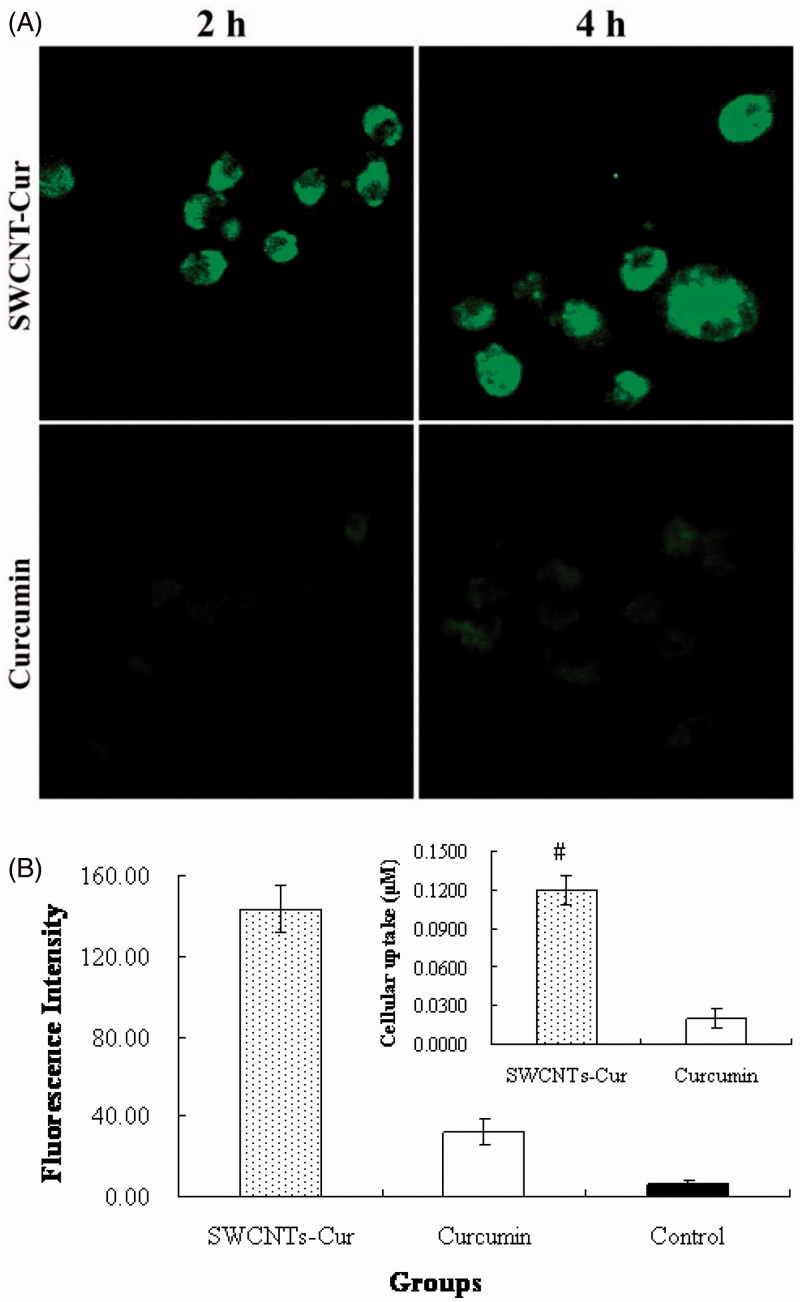
Cellular uptake of 40 μM curcumin (both native and SWCNT-Cur). (A) Confocal images of cells at different times. (B) Quantification of curcumin uptaken by PC-3 cells treated 4 h. ^#^*p* < .05 vs. curcumin group.

Cur can serve as a fluorophore, therefore, the level of intracellular Cur was observed and determined by confocal microscopy and spectrofluorometry. Figure 3(A) showed that a time-dependent increase in cellular uptake of Cur from 2 to 4 h, and it was noteworthy that Cur in SWCNT-Cur was higher uptaken than native Cur within 4 h.

We next quantified the uptake amount of Cur. The result demonstrated that SWCNT-Cur was 6-fold compared to native Cur (Figure 3(B)). This suggests that SWCNT-Cur delivery system can effectively increase the delivery of Cur into cells.

### 3.3. In vivo pharmacokinetic study

**Figure 4.**
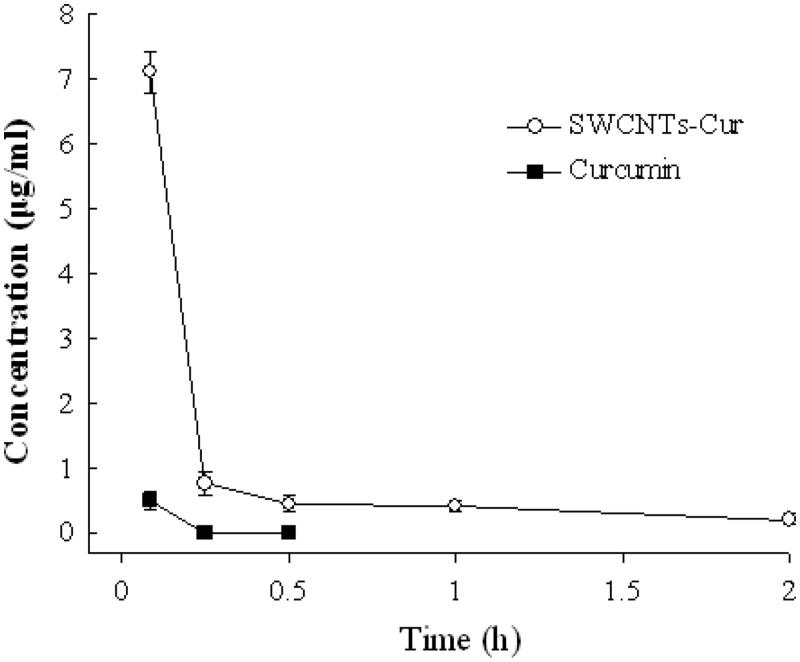
Concentration of curcumin in plasma after intravenous administration of SWCNT-Cur and native curcumin.

Curcumin in plasma achieved 7.10 μg/mL after i.v. injection of SWCNT-Cur at a single dose of 18.8 mg/kg for 5 min, and was still traceable within 2 h. In contrast, the concentration was only 0.39 μg/ml after the same dose of native Cur was injected for 5 min, then rapidly declined to be below detection limit in 30 min (Figure 4). This result suggested that SWCNT-Cur significantly increased Cur’s blood concentration, up to 18-fold, and residence time.

### 3.4. *In vivo* suppression of tumor growth and further photothermal therapy

**Figure 5.**
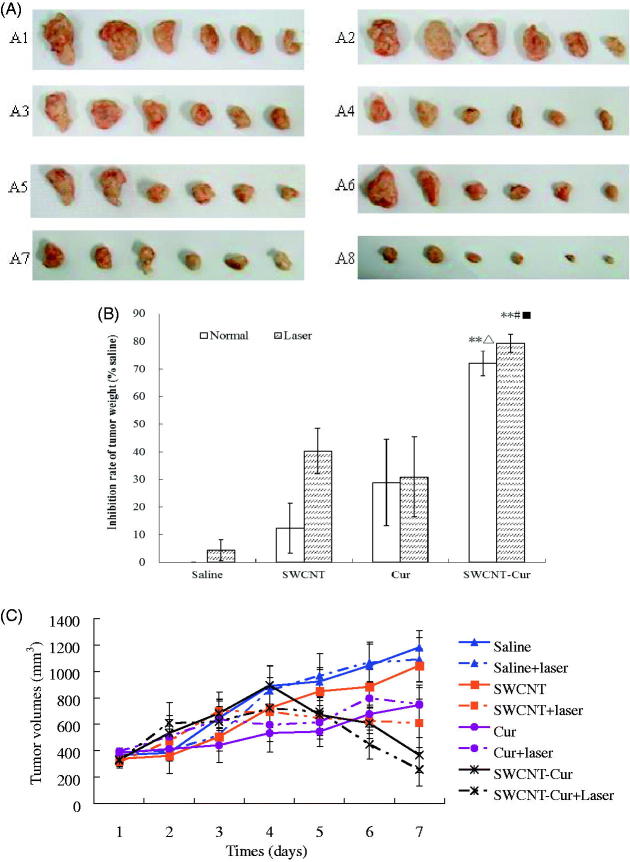
Injection of SWCNT-Cur suppresses S180 solid tumor growth *in vivo*. (A) The tumor photograph after 7 times of administration. A1, control; A2, control + Laser; A3, SWCNT; A4, SWCNT + Laser; A5, curcumin; A6, curcumin + Laser; A7, SWCNT-Cur; A8, SWCNT-Cur + Laser. (B) Inhibition rate of S180 tumor weight *in vivo.* **p < .01 vs. relative control group, ^△^*p* < .05 vs. curcumin group, ^#^*p* < .05 vs. SWCNT group with laser irradiation. ^■^*p* < .05 vs. SWCNT-Cur group without laser irradiation (C) Growth curves of different groups for tumor volume. The SWCNT-Cur group shows significant suppression of tumor growth compared with the control group (*p* < .05).

The results of SWCNT-Cur inhibiting tumor growth *in vivo* are shown in Figure 5. The tumor volume of the saline and saline + laser groups were all getting larger with time passing by, indicating that laser radiation did not inhibit tumor growth. Cur and its laser groups showed certain inhibitory effect compared to the respective saline group, but there was no significant difference between them, indicating that laser did not enhances the antitumor activity of native Cur. And, SWCNT group showed no inhibitory effect, but its laser group did show significant difference compared with the saline + laser group, indicating photothermal ablation efficacy of SWCNT by converting the optical energy into heat energy. Among all the groups, the SWCNT-Cur and its laser groups showed the most significant suppression on tumor weight and volume. SWCNT-Cur’s antitumor effect was not only significantly increased compared to native Cur and SWCNT groups with or without laser irradiation, but also laser irradiation further significantly enhanced tumor suppression activity of SWCNT-Cur (Figure 5(A–C)). The results clearly suggested that synergistic anticancer effect of Cur and photothermal therapy could be obtained by Cur formulated with SWCNT.

### 3.5. Histological analysis

**Figure 6.**
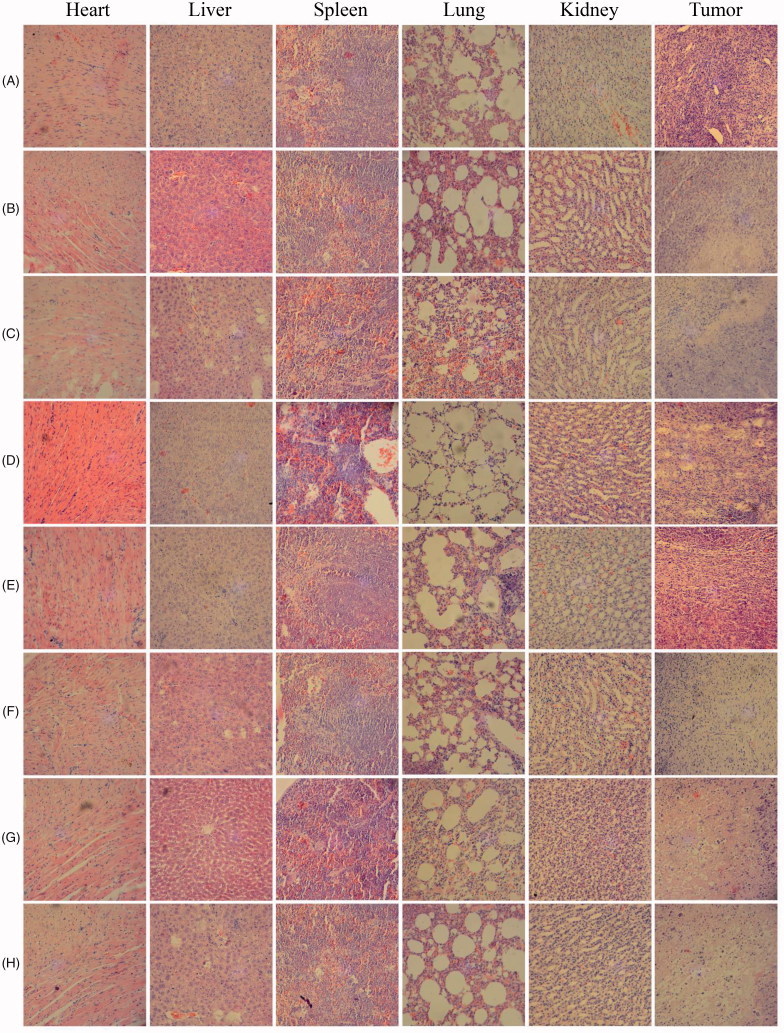
Hematoxylin and eosin staining of heart, liver, spleen, lung, kidney and tumor sections in mice. (A) Control; (B) control + laser; (C) SWCNT; (D) SWCNT + Laser; (E) curcumin; (F) curcumin + Laser; (G) SWCNT-Cur; (H) SWCNT-Cur + Laser.

Results of HE staining in Figure 6 summarized the influence of SWCNT-Cur on the organs of mice. Except for tumor tissues, there were no significant morphological differences among the eight groups for heart, liver, spleen, lung and kidney tissues. However, as for the tumor tissues, control saline group with and without laser showed vigorous growth, tight arrangement, large body and intact shape, while cell necrosis, cell lysis, and cell fragment to a certain extent occurred in all the treatment groups, especially SWCNT-Cur groups, in accordance with inhibition of tumor growth.

## 4. Discussion

SWCNT employed for biomedical purposes is typically in dispersed or functionalized forms to minimize its toxicity (Lanone et al., 2013). In this study, the dispersed and functionalized SWCNT was used, and SWCNT-Cur’s toxicity on the main organs *in vivo* was investigated. In addition, considering methanol served as solution during formulation, we also determined the solvent residue. The results showed that the obvious toxicity and adverse effects were hardly observed in SWCNT-Cur formulation and content of methanol is far less than the pharmacopeia requirements, suggested from the result of GC chromatogram and HE staining of heart, liver, spleen, lung, and kidney of mice (Figures 1 and 6). It is thus clear that the SWCNT-Cur used in this study is biocompatible.

The physicochemical characterization of drug in delivery systems influences its stability, cellular uptake and plasma concentration (Aqil et al., 2013), which are all vital for bioaccessibility of drug (Blanco et al., 2013; Zheng et al., 2018). Therefore, researchers have constantly developed different formulations of Cur to circumvent its physicochemical limitations for enhancing bioavailability (Jamwal, 2018; Zheng et al., 2018; Saheb et al., 2019; Wong et al., 2019). Previously, we observed a significant increase of Cur’s stability and solubility in SWCNT-Cur (Li et al., 2014a). In this study, the difference of physicochemical characterization between native and formulated Cur was also clearly demonstrated by IR spectra (Figure 2). After loading into functionalized SWCNT, hydrogen bonds were formed between Cur and the carriers, suggesting from the change of Cur’s characteristic peaks, which may be one of reasons of the improvement of Cur’s stability and solubility. Furthermore, the enhanced cellular uptake of Cur was achieved by the present SWCNT carriers system (Figure 3). Importantly, the poor plasma concentration of Cur, which does not reach the required level of exerting its anticancer effect and is major obstacles limiting its applications (Jamwal, 2018; Chen et al., 2019; Sahne et al., 2019), has been dramatically improved, up to 18-fold, by the present delivery system (Figure 4). Therefore, the enhanced stability, solubility, cell uptake and the high plasma concentration of Cur together resulted in the adequate Cur delivery to tumors. Thus, SWCNT-Cur enhanced Cur’s bioaccessibility.

Combination therapy of cancer is becoming more popular because it generates synergistic anticancer effects or reduces individual drug-related toxicity through different mechanisms of action (Lin, et al., 2018; Al Fatease et al., 2019; Tan & Norhaizan, 2019). By virtue of the high optical absorbance at 808 nm, SWCNT can act as photothermal therapy agents for ablating cancer cells due to excessive local heating (Robinson et al., 2010), which is different from the Cur. Thus, in the present study, SWCNT served not only as scaffolds, but also as thermal ablation agent, to further synergistically enhance Cur’s antitumor effect (Figure 5). In addition, Cur exerts beneficial effects on cancer treatment-related neurotoxcity, cardiotoxicity, nephrotoxicity, hemato-toxicity, and so on (Willenbacher, et al., 2019), which may be also benefit for SWCNT’s biocompatibility.

**Figure 7.**
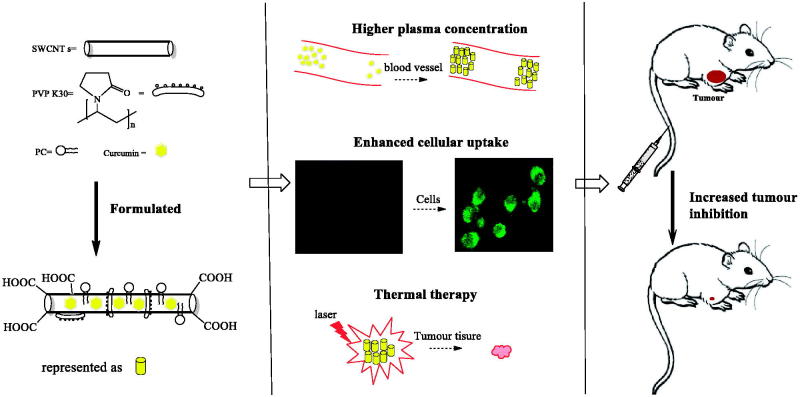
Summary of the mechanisms by which SWCNT-Cur more inhibits tumor growth than curcumin. SWCNT-Cr enhanced the delivery of curcumin into tumor cells, improved the blood level of curcumin and further ablated the tumor growth via SWCNT induced photothermal effect. All of above mentioned aspects synergistically enhanced anti-tumor of SWCNT-Cur.

In conclusion, SWCNT-Cur exhibited more efficient anti-tumor activity than Cur and SWCNT’s photothermal ablation. The mechanism underlying this effect may include three aspects (Figure 7): (1) SWCNT-Cur enhances the delivery of Cur into tumor cells by enhancing cellular uptake. (2) SWCNT-Cur increases the plasma level of Cur by improving Cur’s physicochemical characterization. (3) Thermal therapy mediated by SWCNT further facilitated SWCNT-Cur to ablate the tumor growth. Thus Cur delivery with functionalized SWCNT is a promising strategy for combination therapy to enhance anticancer activity *in vivo.*
